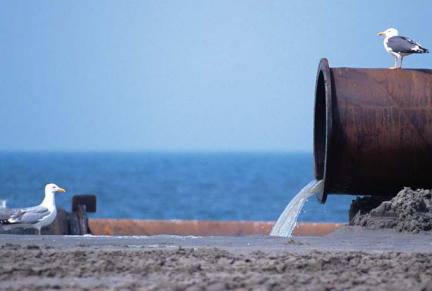# Chemical Exposures: An Eye to the Sea

**DOI:** 10.1289/ehp.116-a156

**Published:** 2008-04

**Authors:** Graeme Stemp-Morlock

Gardening, choosing contraception, driving to work—we don’t typically think of these actions as having a high impact on fisheries, but they can. A session titled “From Kitchen Sinks to Ocean Basins: Emerging Chemical Contaminants and Human Health” at the February 2008 annual meeting of the American Association for the Advancement of Science detailed some recent findings showing that certain day-to-day activities can have big impacts on the oceans.

Think of an oil spill like the 1989 *Exxon Valdez* incident, and you may visualize images of wildlife blanketed in black crude, struggling against acute poisoning and hypothermia. But John Incardona, a scientist with the National Oceanic and Atmospheric Administration (NOAA), was surprised that some spill compounds could cause far less obvious, though equally dramatic, effects. He found that polycyclic aromatic hydrocarbons (PAHs) caused heart defects in fish after the *Exxon Valdez* spill. He also found that PAHs had a similar adverse response in zebrafish, which is in many ways a better model of the human heart than the more commonly used mouse or rat.

PAHs are also released during the combustion of wood, coal, and oil. “The emissions from a tailpipe include the PAHs created by burning but also aerosolized fuel that doesn’t get burned,” said Incardona. “In essence, the air is just like an aerosolized oil spill. And, if a physician knowingly gave an aerosolized cardiotoxicant to a patient with coronary artery disease or congestive heart failure, they would probably be sued for malpractice. But the air in big cities is doing just that to millions of people every day.”

Untested synthetic chemicals emitted into waterways also could put fisheries at risk. Derek Muir, an environmental chemist with Environment Canada, wanted to find out if any of the 22,000 chemicals in medium to high levels of production today are as bioaccumulative or persistent as banned substances such as DDT and polychlorinated biphenyls. He developed a computer model that analyzed compounds’ structures and estimated levels of persistence using a database of known bioaccumulation rates. He found 400 chemicals that fit the persistence bill. Of those 400, 75% have never been described in the environmental literature, only 4% are regularly analyzed, and all have been in production for several decades.

Other research focused on 17α-ethinyl-estradiol (EE_2_), a synthetic estrogen used in women’s birth control. EE_2_ passes through the female body and, later, wastewater treatment plants, often finding its way into fish when treated water is discharged into nearby rivers, streams, and oceans. Several studies in recent years have described the feminization of male fish in response to estrogenic exposures, but Karen Kidd, an ecotoxicologist with the University of New Brunswick, presented some of the first findings on how estrogens could affect an entire population.

She exposed an isolated test lake in northwestern Ontario to 5 ng/L EE_2_, about average for streams and rivers receiving wastewater effluent. After one summer, male fish began releasing egg-forming proteins and had delayed sperm development, while females’ sexual development was significantly delayed. As a result, the fathead minnow in the lake stopped reproducing, and their population collapsed. That collapse had profound implications for larger predators such as lake trout, whose own population dropped 30% as a result.

“These results are sobering because they show that estrogens can affect fish abundances both through direct toxicity or indirectly through the food chain because of the loss of their food supply,” said Kidd. EE_2_ can be easily broken down, however, and improvements to wastewater processing could mean less estrogen makes it into waterways. [For more on one possible improvement, see “Fe-TAML Takes On Estrogens in Effluent,” p. A159 this issue.]

Finally, although the effects of many chemicals are known, the way they interact with other chemicals is not always clear. “We’ve got a pretty good handle on how to assess the health effects of single chemicals in toxicity trials,” said NOAA zoologist Nathaniel Scholz. “But the real world is more complex, and exposure to multiple chemicals in mixtures is the rule.”

Using salmon as a test model, Scholz found that combinations of pesticides that run off into river waters frequently were synergistic, and certain combinations (such as diazinon plus malathion, both common pest control chemicals) can be lethal. This research is critical to the success of the multimillion-dollar effort to restore salmon habitat on the West Coast. As well, it may shed light on human health effects, because the human nervous system is similar to that of the salmon. “Pesticide residue typically occurs in the human food supply at very low levels, but pesticide mixtures are also very common,” Scholz said. “This creates the potential for synergistic interactions and enhanced toxicity to people.”

## Figures and Tables

**Figure:**